# Prevalence and risk factors for Bovine Herpesvirus Type 1 (BoHV-1) infection in Irish beef herds: results from the National Beef Welfare Scheme 2023

**DOI:** 10.1186/s13620-025-00308-0

**Published:** 2025-09-29

**Authors:** Jonas Brock, Maria Guelbenzu-Gonzalo, Jose Maria Lozano, Elizabeth A. Lane, Michael Gunn, Sean Brady, Hans-Hermann Thulke, David A. Graham

**Affiliations:** 1 Animal Health Ireland, Carrick-On-Shannon, Co. Leitrim Ireland; 2 Helmholtz Centre for Environmental Research GmbH - UFZ, PG Ecological Epidemiology, Leipzig, Germany; 3 Department of Agriculture, Food and the Marine, Virology Division, Backweston laboratories, Virology Division, Celbridge, Republic of Ireland; 4 Department of Agriculture, Food and the Marine, One Health One Welfare Scientific Support Team, Agriculture House, Kildare St, Dublin 2, Ireland; 5 Anvil Consulting Services Limited, Ballynahinch, County Down, Northern Ireland

**Keywords:** IBR, BoHV-1, Cattle, Beef, Ireland, Prevalence, Eradication, NBWS

## Abstract

Infectious bovine rhinotracheitis (IBR), caused by bovine herpesvirus-1 (BoHV-1), is a highly contagious disease with significant economic impacts on the cattle industry. It can also lead to respiratory distress, reproductive losses and compromised animal welfare, and thus represents a key target for control. This study aimed to assess the prevalence and identify risk factors associated with BoHV-1 infection in Irish beef herds. Conducted under the National Beef Welfare Scheme (NBWS), the study involved testing 10,659 beef breeding herds, representing approximately 20% of the national beef herd population. A total of 189,404 animals were tested. Using a ‘snapshot’ testing strategy herd-level BoHV-1 status was determined based on the presence of antibodies to the gE glycoprotein in up to 20 randomly selected animals, preferably over 9 months of age to exclude maternally derived antibodies. Vaccination histories were not available for participating herds. Results indicated an animal-level apparent prevalence of 11.4% and a herd-level apparent prevalence based on positive snapshots of 48.8% (defined as herds with ≥ 1 positive animal). Larger herds and high rates of animal in-moves per capita (here, > 17% of herd replaced by purchases in the past year) were identified as significant risk factors for recent (within the last three years) BoHV-1 circulation. Previous studies had indicated a herd-level prevalence in Ireland of up to 80%. The lower prevalence estimates identified in this study may reflect improved biosecurity and vaccination uptake in recent years. The findings from this survey, although showing that BoHV-1 is still endemic in Irish beef herds, provide updated prevalence figures which are considerably lower, indicating that a higher number of farms would be in a position to achieve freedom from BoHV-1 in a relatively short period. These results offer essential epidemiological insights to inform the design and implementation of a national BoHV-1 control programme in Ireland.

## Introduction

IBR is a highly infectious disease caused by BoHV-1 that not only imposes substantial economic losses through reduced production and increased veterinary costs, but also contributes to respiratory distress, reproductive failure and overall compromised animal welfare [1–4]. The virus typically spreads by close contact between animals although airborne transmission may occur over distances of up to 5 m and it can also be spread by using contaminated semen, and indirectly by equipment and people [4]. Once an animal becomes infected, it remains infected for life despite developing immunity [4]. The virus establishes a lifelong latent infection in the nerve cells within the animal’s central nervous system. During this period the latent carrier is not shedding virus. However, at times of stress, such as transport, mixing of stock, calving, nutritional stress and inter-current disease, the virus may be reactivated and can begin to multiply and be re-excreted, generally in nasal and ocular secretions [4–6]. This can lead to new infection and disease in other susceptible cattle, which in turn will also become latent carriers [4]. These latently infected carriers play a central role in maintaining BoHV-1 in infected herds, where they act as a reservoir of infection, and in spreading infection between herds.

In Ireland, infection with BoHV-1 is widespread in both dairy and beef herds with previous studies having found evidence that 75–80% of herds have been exposed to BoHV-1 and contain carrier animals [7–10]. One study estimated the mean within herd prevalence (the mean proportion of seropositive carrier animals per herd) in Irish beef herds to be 40% and found a positive association between seroprevalence to BoHV-1 and the herd size and mortality rate [10]. Although our focus is on beef herds, previous dairy herd estimates offer useful national benchmarks for BoHV-1 seroprevalence and facilitate comparison across production systems. A study of Irish dairy herds found a within herd seroprevalence in BoHV-1-positive, unvaccinated dairy herds of 31.7% [11]. Regarding the most relevant factors for introduction of BoHV-1 into cattle herds, a recent literature review found that these include herd size, purchase of cattle, cattle density, age of cattle, distance to neighbouring cattle herds and professional visitors [12].

In Ireland, beef breeding herds (commonly called suckler herds) represent the largest cattle enterprise, with nearly 50 000 registered herds in 2019 [13]. Five subtypes reflect the progression from birth to sale or slaughter: Beef Suckling to Weanling (BSW), Beef Suckling to Youngstock (BSY), Non-rearing BSY (BSY-nR), Suckling to Beef (BSB), and Beef Pedigree (BP). BSW herds rear calves to weaning (September–October sales), while BSY herds retain calves up to 12–20 months. BSY-nR herds sell female calves at weaning, replacing breeders by purchase; BSB cover the full cycle to slaughter; and BP herds supply pedigree breeding stock [13]. Data on IBR vaccine usage specifically in beef herds are lacking, although national vaccine sales have risen in recent years.

The economic losses associated with BoHV-1 can be substantial, with infected herds experiencing reduced milk yields, increased culling rates, and higher veterinary costs [2, 14, 15]. In Ireland, farmers are estimated to be spending around 10 million Euros annually on BoHV-1 vaccines, highlighting the significant financial burden of the disease.

At the time of writing, the establishment of a national BoHV-1 control programme is being considered in Ireland, in line with the efforts of many European countries that have already implemented successful BoHV-1 control programmes. Several countries or regions within the European Union (EU) are considered free from BoHV-1 following the implementation of EU-approved eradication programmes. These include Austria, Germany, Denmark, Finland, Sweden, Czechia and the Italian regions of Valle d'Aosta and the Province of Bolzano. As a result, these countries/regions are granted additional trade guarantees. Other EU countries and regions, such as Belgium, Luxembourg, multiple regions of France and the Italian provinces of Friuli-Venezia Giulia and Trento, are currently implementing Commission-approved BoHV-1 eradication programmes [16, 17]. The BoHV-1 trade restrictions and additional guarantees for BoHV-1-free countries/regions impact the trade of live cattle from countries or regions that are not free from the disease, particularly when there are not an approved eradication programmes in place.

A national programme to control BoHV-1 does not currently exist in Ireland, but is under active discussion, co-ordinated by Animal Health Ireland (AHI; www.animalhealthireland.ie), a not-for-profit public–private partnership established to improve the profitability and sustainability of the Irish farming and agri-food sector. This study was specifically designed to inform those national-level discussions and to support stakeholders in assessing the feasibility, structure, and strategic targeting of a future BoHV-1 control or eradication programme in Ireland.

Understanding the prevalence and epidemiology of BoHV-1 is crucial for the development of effective control and prevention strategies. The objective of this study was to conduct a descriptive analysis of BoHV-1 infection in Irish beef cattle herds, providing valuable insights into the status of the disease in the country, and to perform a risk factor analysis to identify herd characteristics that explain recent within-herd BoHV-1 circulation. Given that earlier seroprevalence studies in Irish beef herds were smaller in scale and are now outdated, and that national IBR vaccine uptake has increased recently, these updated figures—covering approximately 25% of all beef herds—are essential to inform the planning of a potential eradication programme.

## Methods

### Data and testing

This study was conducted as part of the National Beef Welfare Scheme (NBWS) in 2023, a government-funded and voluntary programme in Ireland that aims to further increase the economic efficiency of and enhance animal health and husbandry on suckler farms. The NBWS provided financial support for the testing of cattle herds for BoHV-1 antibodies. In total 10,659 beef breeding herds participated in the testing programme, which is approximately 20% of the entire Irish beef herd population [13].

To determine the herd-level BoHV-1 status, a'snapshot'test was performed using a blood sample from up to 20 randomly selected animals from each herd, ideally 9 months old or above to avoid maternally derived antibodies, or all ages if there were fewer than 20 on the holding on the day of testing. Where a herd had twenty or more bovines, a minimum of twenty were to be tested and where a herd had less than twenty bovines, all were tested. Sampling took place between 3rd August 2023 and 20th December 2023, thus covering the late summer to early winter period. While guidance was provided to encourage random selection of animals, all sampling was carried out by local practicing veterinarians. As the study authors were not directly involved in the sampling process, we cannot confirm the extent to which true randomisation or systematic selection procedures were implemented in practice.

The decision to sample a maximum of 20 animals per herd was made by the Department of Agriculture, Food and the Marine (DAFM), who administered the NBWS. The study authors were not involved in determining this sampling threshold. While no formal sample size calculation was shared with the authors, it is understood that the 20-animal limit was determined primarily.

The collected samples were submitted to one of the laboratories listed in the Department of Agriculture, Food and the Marine’s (DAFM) Terms and Conditions for the NBWS. All participating laboratories were ISO17025 accredited and used commercially available gE ELISA kits. While specific kit information was not systematically linked to individual test results, the list of accredited assays includes the IDEXX gE Competition ELISA, IDvet SCREEN ELISA, and the Indical Cattletype BHV-1 gE ELISA. All tests were applied and interpreted according to the respective manufacturers’ thresholds for sample-to-negative (S/N) ratios.

The gE ELISA was selected because it enables differentiation between wild-type BoHV-1 infection and vaccination with marker (gE-deleted) vaccines. Since 2004, only marker vaccines have been authorised for use in Ireland [18], making the gE test appropriate for identifying field virus exposure. As individual vaccination records for participating herds were not available, this test provided the most reliable estimate of field infection prevalence under the given conditions.

On receipt of samples, the laboratories recorded the identity of each animal sampled in the Irish Cattle Breeding Federation (ICBF) database in each herd in advance of testing of the samples. A sample-to-negative (S/N) ratio was calculated for each test, and the numeric value was classified into interpreted results of positive, negative, or inconclusive depending on the manufacturers’ thresholds. The herd-level BoHV-1 status was determined based on the presence of BoHV-1 antibodies in the tested animals. Following testing, results were uploaded to the ICBF database where they were accessible to herd owners. Herds were classified as BoHV-1 positive if at least one animal within the herd tested positive for BoHV-1 antibodies. For the following analysis, inconclusive results were considered negative. Furthermore, only herds with either complete results or a single missing test result were included in the analysis. This was the case when all expected test results were available for a herd (up to 20 tests depending on herd size), or if only one result was missing, based on a comparison of the number of samples initially logged for each herd against the number of results that were subsequently uploaded. The rationale for including herds with a single missing result—but excluding those with more—was based on a review of the distribution of missing test data across herds and consultation with an expert panel overseeing the analysis.

### Descriptive analysis

A descriptive analysis of the BoHV-1 antibody data was conducted to provide a comprehensive understanding of the status of the disease within the tested cattle herds.

To assess the extent to which results could be considered representative of the national population, the representativeness of the herds tested was assessed by comparing the distribution of herd types (as described in [13]) and herd sizes in the NBWS herds to the distribution of all beef breeding herds in Ireland. For this analysis, herd demography data for all registered beef breeding herds was obtained from the Animal Identification and Movement (AIM) national database maintained by the DAFM in Ireland. The intention of this analysis was to ensure that the results could be extrapolated to the national level.

In a second step, the herd- and animal-level apparent prevalences were calculated, along with their respective 95% confidence intervals. The animal-level prevalence calculation process involved dividing the number of animals testing positive for BoHV-1 antibodies by the total number of animals tested. Similarly, the herd-level prevalence was determined by considering the presence of at least one animal testing positive for BoHV-1 antibodies within a specific herd. The corresponding 95% confidence intervals (CIs) were calculated using the exact Clopper–Pearson method, which is appropriate for binomial proportions.

### Risk factor analysis

To identify potential risk factors associated with recent (within the last three years) BoHV-1 antibody positivity, a case–control study at the herd level was conducted in accordance with the guidelines laid down in the STROBE statement [19, 20].

Cases were defined as herds that had serological evidence of active BoHV-1 circulation within the past three years, while control herds were those that tested negative for BoHV-1 antibodies. The three-year timeframe was selected pragmatically by the study team, with input from the expert panel overseeing the analysis. This threshold was not based on a specific published framework but was chosen to focus on more recent virus circulation, which is most relevant for informing control and eradication strategies. The intention was to exclude historical infections that may no longer reflect the current epidemiological risk in herds.

To determine whether a herd had active BoHV-1 circulation in the past three years, a filtering process was applied to all seropositive herds. The age of the youngest homebred positive animal within each herd was calculated to approximate the time since the last active virus circulation. This approach was based on the following theoretical framework.

Previous studies indicate that once BoHV-1 is introduced into a herd, it is likely to spread and affect all animals on the farm, resulting in seropositivity across all age cohorts [11]. Infected animals remain antibody-positive for life, serving as indicators of past virus circulation. As viral circulation ceases, newborn animals will be seronegative. By assessing age-dependent seroprevalence patterns, step-wise increases in seropositivity can be observed within herds, as described by [11] This pattern allows the approximation of the timing of the last active BoHV-1 circulation within a given herd. By employing this methodology, herds were classified accurately into case and control groups, providing a reliable basis for further analysis of potential risk factors associated with recent BoHV-1 circulation.

An initial univariable analysis was performed to explore the distribution of variables and visually examine differences between case and control herds using boxplots and bar charts. Statistical comparisons between cases and controls were also conducted. Throughout, linear predictors were categorised based on quartiles. This was done to accommodate non-normal distributions and to meet the assumptions of the logistic regression model, while also facilitating interpretation of the effect sizes across different value ranges. The chi-squared (χ2) test of independence was employed to determine whether there is a statistically significant relationship between each of the selected herd characteristics and the herd's BoHV-1 status. The variables analysed in the univariable analysis included: herd size (at the time of the test), herd type, number of animal movements into the herd per capita and proportion of non-homebred animals in the herd. All risk factor data were sourced from the Animal Identification and Movement (AIM) database, Ireland’s national cattle registry maintained by the Department of Agriculture, Food and the Marine. After checking for variable collinearity using the variance inflation factor with a threshold of 5 applied to identify potentially collinear variables, a multivariable logistic regression model was trained on the data using a stepwise backward variable selection approach. The backward variable selection approach was based on Akaike’s Information Criterion (AIC). The results of the final model were presented as odds ratios with corresponding 95% confidence intervals, indicating the strength and direction of the association between each risk factor and recent BoHV-1 circulation.

All statistical analyses and data visualisations were conducted using R software.

## Results

### Herds and animals included

Blood samples were received for 10,659 beef herds and individual test results were available for 188,796 animals. Of the 10,659 herds for which results are available, 6,455 herds (60,5% of all NBWS participating herds) had complete records or only a single result missing. For the remaining 4,204 herds at least two results were missing. In the following analysis, data for the 6,455 herds with complete or nearly complete data is presented and are referred to as ‘study herds and animals. In these herds a total of 126,028 individual tests results are available.

### Representativeness of study herds and animals

**Fig. 1:**
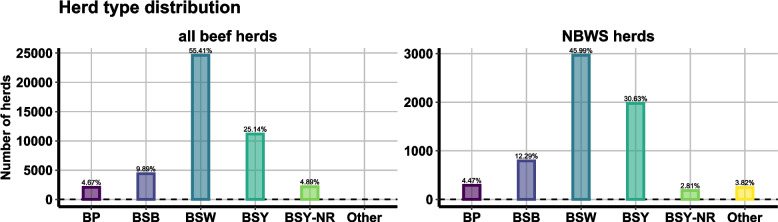
Herd type distribution. The figure compares the distribution of herd types in NBWS-participating herds with all registered beef breeding herds in the Republic of Ireland at the time of sampling (data from the AIM database). Five different subtypes of beef herds have been identified in Ireland, which are consistent with recognised production systems which differ in their management characteristics including when animals are sold for further production. Beef suckling to weanling (BSW) producers maintain a herd of cows and raise calves from birth to weaning, with the majority sold as weanlings at autumn sales during September and October while a proportion of female calves is kept as heifer replacements. The beef suckling to youngstock (BSY) subtype is similar to BSW, including retaining a proportion of females as replacements, with the key difference being that calves are kept for a longer period, to allow weaned calves to gain weight prior sale. These animals are usually yearlings (12–20 months of age) by the time they leave their birth herd. Non-rearing suckling to youngstock (BSY-NR) herds are a variation of the BSY herd type, with the difference that female calves are sold after weaning and replacement bred females are purchased. The suckling to beef (BSB) herds follow the full beef production cycle, from birth through to the age of slaughter. Finally, representing only a small proportion of the beef sector in Ireland, beef pedigree (BP) herds are an important source of pedigree breeding stock to other commercial cattle producers in both the dairy and beef sectors

The herds surveyed in the NBWS appear to be a representative subset of all beef breeding herds in Ireland in terms of herd type composition. Figure 1 shows the distribution of herd types in NBWS herds in comparison with the distribution of all beef breeding herds in Ireland. Beef suckling to weanling (BSW) herds, which maintain a herd of cows and raise calves from birth to weaning, are by far the most common herd type in both populations.

**Fig. 2:**
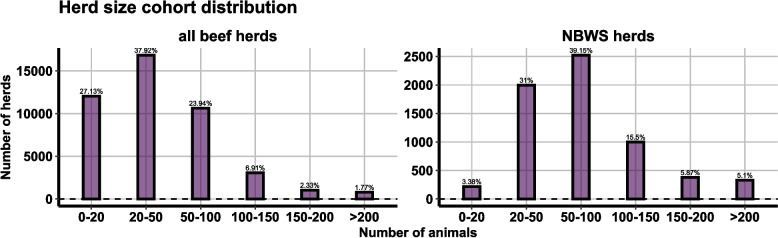
Comparison of herd size cohort distribution

While the overall distribution of herd types is broadly consistent between NBWS herds and all beef herds, NBWS herds tend to be larger on average (mean herd size: all beef herds—49.2 animals vs. NBWS herds—81.7 animals). This is mainly due to small herds (0–20 animals) being underrepresented in the NBWS sample (see Fig. 2).

**Fig. 3:**
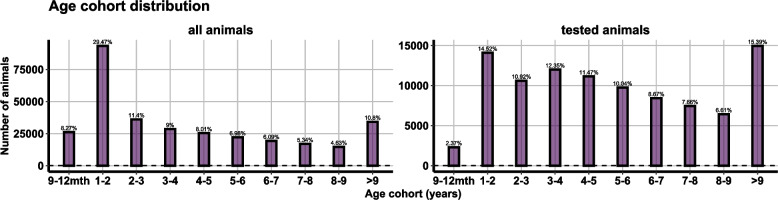
Comparison of age distribution of all animals in tested herds versus tested animals including only animals over 9 months old

Figure 3 provides a comparison between the age distribution of all animals in the tested herds at the time when the herd was sampled in comparison to the age distribution of those tested. Upon excluding the very young animals (0–9 months old) from the comparison due to the applied sampling strategy, the age distribution of the tested animals in the NBWS aligns closely with the age distribution of all animals in the tested herds. However, a slight age discrepancy between the groups can be observed, which is also represented in the average age-figures. The average age of tested animal was 5 years, whereas the average age of all animals in the herd at time of testing was 3 years.

### Prevalence estimation

Among the 126,028 study animals, 14,371 returned a positive outcome, resulting in an animal-level apparent prevalence of 11.4% (95% CI: 11.2%—11.6%) (see Table 1). The majority of animals were negative (87.8%), while 0.8% yielded inconclusive results. At the herd level, our data indicated a herd-level apparent prevalence of 48.8% (95% CI: 47.6%—50.0%).

**Table 1:** Animal- and herd-level apparent prevalence

*Animal-level results*		
***BoHV-1 animal status***	**Number of animals**	**Proportion**
*(Sample)-Negative*	110,592	87,8%
*(Sample)-Positive*	14,371	11,4%
*(Sample)-Inconclusive*	1,065	0.8%
*Total*	126,028	

*Herd-level results*		
*BoHV-1 herd status*	Number of herds	Percentage
*(Sample)-Negative*	3,056	47.3%
*(Sample)-Positive*	3,150	48.8%
*(Sample)-Inconclusive*	249	3.9%
*Total*	6,455	

**Fig. 4:**
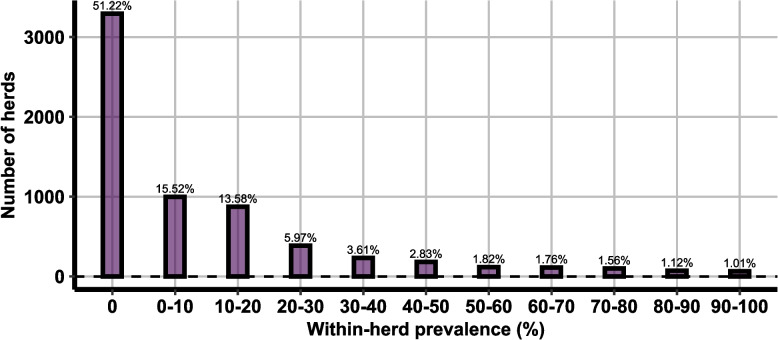
Snapshot within-herd prevalence (% of samples testing positive per herd)

For the 6,445 study herds, the distribution of the snapshot within herd prevalence is shown Fig. 4. In 51.2% of tested herds no positive animal was detected. In a further 15.5% of study herds, the snapshot within-herd prevalence was < 10%.

### Age-cohort of the youngest positive animal

**Fig. 5:**
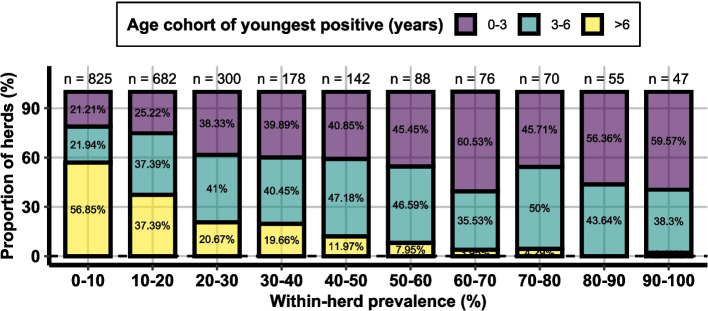
Stacked bar chart showing the age-cohort of the youngest positive animal per herd, structured by within-herd prevalence group

For all positive herds (*n* = 3,150), Fig. 5 presents the age cohort of the youngest positive homebred animal, categorised by within-herd prevalence groups. In low-prevalence herds (e.g., < 10%), infections are predominantly found in older animals. Specifically, in 57% of these low-prevalence herds, the youngest positive animal is older than six years.

### Herd-size related prevalence

**Fig. 6:**
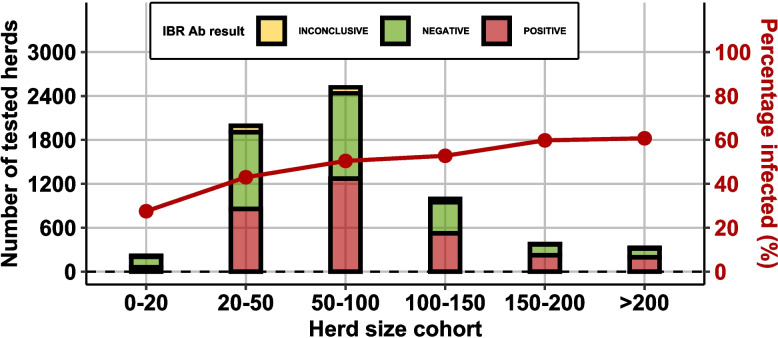
Proportion of seropositive herds by herd size

For the study herds, the proportion of infected herds by herd size was plotted as seen in Fig. 6. A clear size-dependent trend can be observed in the data (e.g. 60% of herds with 200 or more animals returned at least one positive test).

### Proportion of BoHV-1 seropositive study herds by county

**Fig. 7:**
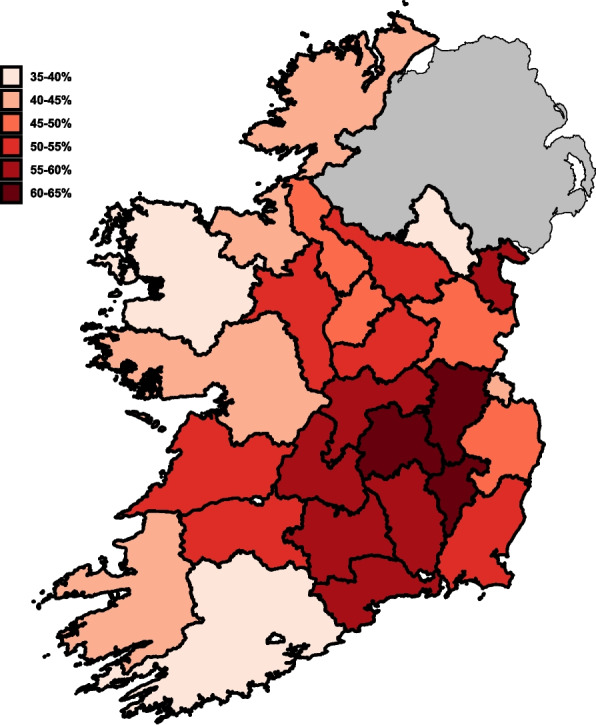
Proportion of BoHV-1 positive study herds (in relation to all tested herds) by county

Figure 7 and Table 2 show the proportion of BoHV-1 positive study herds by county. Depending on county, the proportion of BoHV-1 positive herds among the tested herds lies between 35.7% (Co. Monaghan) and 63.1% (Co. Carlow).

**Table 2:** Apparent prevalence by county

County	Tested herds	Positive herds	Prevalence in % (95% CI)
Carlow	103	65	63.1 (53.4—71.8)
Laois	198	122	61.6 (54.5—68.2)
Kildare	62	38	61.3 (48.4—72.6)
Tipperary	339	202	59.6 (54.3—64.9)
Waterford	110	65	59.1 (50.0—68.2)
Offaly	137	78	56.9 (48.9—65.0)
Kilkenny	171	96	56.1 (48.5—63.7)
Louth	40	22	55.0 (40.0—70.0)
Wexford	193	105	54.4 (47.2—61.7)
Westmeath	212	115	54.2 (47.6—60.8)
Roscommon	401	214	53.4 (48.4—58.4)
Clare	562	296	52.7 (48.6—56.8)
Limerick	201	106	52.7 (45.8—59.7)
Cavan	261	132	50.6 (44.4—56.7)
Meath	153	76	49.7 (41.8—57.5)
Longford	222	107	48.2 (41.4—55.0)
Leitrim	168	81	48.2 (40.5—56.0)
Wicklow	94	45	47.9 (38.3—57.4)
Galway	757	339	44.8 (41.2—48.3)
Donegal	240	105	43.8 (37.5—50.0)
Dublin	14	6	42.9 (21.4—71.4)
Kerry	327	139	42.5 (37.3—48.0)
Sligo	260	109	41.9 (35.8—48.1)
Mayo	510	204	40.0 (35.7—44.3)
Cork	511	204	39.9 (35.6—44.2)
Monaghan	199	71	35.7 (29.1—42.2)

### Risk factor analysis

The risk factor analysis identified two key variables significantly associated with recent BoHV-1 circulation in Irish beef herds. The final logistic regression model included herd size and the proportion (%) of animal in-movements per capita (Table 3). Both of the retained variables showed a significant correlation with the likelihood of a herd experiencing recent virus circulation.

**Table 3:** Conditional associations between retained predictor variables and infection status

Variable	OR (95% CI)	*P*-value
Herd size cohort		
Q1 (Herd size: 1—41)	Referent	
Q2 (Herd size: 42—65)	1.00 (0.77–1.29)	0.97
Q3 (Herd size: 66—102)	1.31 (1.02–1.66)	< 0.05
Q4 (Herd size: > 102)	1.73 (1.36–2.21)	< 0.05
In-moves per capita cohort		
Q1 (In-moves per capita: 0%—2.6%)	Referent	
Q2 (In-moves per capita: 2.7%—7.8%)	1.59 (1.22–2.07)	< 0.05
Q3 (In-moves per capita: 7.9%—16.9%)	2.36 (1.83–3.06)	< 0.05
Q4 (In-moves per capita: > 16.9%)	3.05 (2.37–3.95)	< 0.05

The analysis revealed a strong positive association between herd size and recent BoHV-1 circulation. Herds in the largest size cohort (greater than 102 animals) had the highest odds of recent active virus circulation (OR = 1.73, 95% CI: 1.36–2.21) compared to the smallest herds (1–41 animals), which served as the reference category.

The proportion (%) of animal movements into a herd (in-moves) per capita was the other significant risk factor (Table 3). Herds in the highest quartile of in-moves per capita (more than 16.9%) were more than three times as likely of having recent virus circulation (OR = 3.05, 95% CI: 2.37–3.95) compared to herds with the fewest in-moves per capita (0%−2.6%), which served as the reference group.

**Fig. 8:**
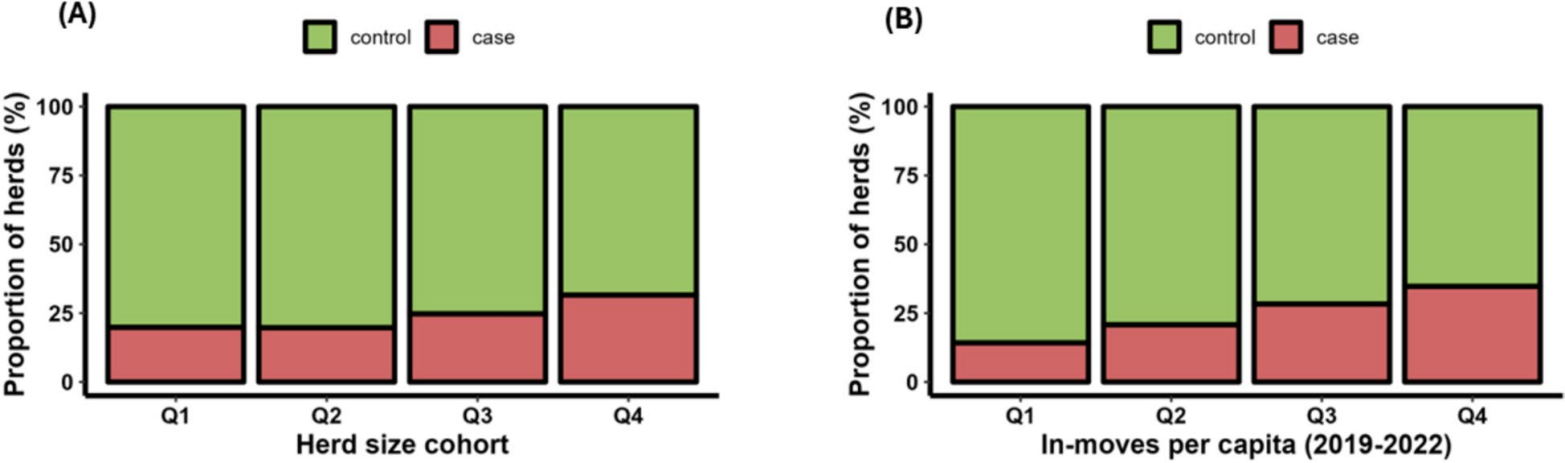
Univariate plots showing the relationship between the BoHV-1 antibody status and each retained predictor. **A** Herd Size Cohort. **B** In-move per capita cohort. The value ranges for each quantile can be seen in Table 3

For the two variables retained in the final model, univariate plots were generated to illustrate the relationship between the outcome (recent BoHV-1 circulation) and each predictor variable individually (Fig. 8).

The final logistic regression model demonstrated good overall fit, with a residual deviance of 3045.4 on 2887 degrees of freedom and an AIC of 3059. The distribution of deviance residuals did not indicate major violations of model assumptions (range: –1.04 to + 2.06). The model’s area under the ROC curve (AUC) was 0.74, indicating modest discriminatory ability to distinguish between herds with and without recent BoHV-1 circulation.

## Discussion

### Herd and animal apparent prevalence

**Fig. 9:**
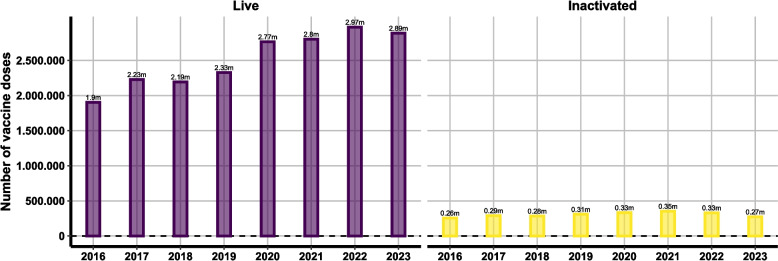
Number of doses of BoHV-1 vaccine sold per year per vaccine type (inactivated, live). Data from Kynetec

In the context of the Irish beef cattle sector, BoHV-1 remains endemic albeit at a lower prevalence than previously reported. While previous studies reported notably high prevalence rates (e.g. [7–10]) the current herd-level prevalence of 49% reflects a decrease from previous findings. This decline in prevalence may be attributed to various factors such as improved biosecurity measures, vaccination strategies, or changes in farming practices that have contributed to a reduction in BoHV-1 transmission within herds. Currently in Ireland there is a continued high level of expenditure on BoHV-1 vaccination. During 2022, analysis of sales data indicates that over 3.3 million BoHV-1 vaccine doses were sold (Fig. 9). This was an increase from the previous 12 months and reflects a continuing trend seen over the past seven years, although sales dropped slightly in 2023, possibly as a reflection of lack of availability.

A previous Irish study of 6,000 beef suckler cows described an association between the presence of BVDV antibody positive animals and seroconversion to BoHV-1 [10]. This could be due to the immunosuppressive effects of BVDV and would suggest that the ongoing BVDV eradication efforts could be assisting in reducing the seroprevalence of BoHV-1. However, more recently, a study that included results from an abattoir survey from 2018 and 2020 of over five thousand animals under 30 months, found that 21% were seropositive to BoHV-1 [21]. The relationship with BVDV serological status was also explored, but the association was not sufficiently robust.

In our study we found a substantial proportion of positive herds (30%) with within-herd prevalence values below 20% (Fig. 4). The low prevalence suggests that targeted interventions in these herds could lead to rapid containment and potentially facilitate the eradication of BOHV-1 on a broader scale. A more detailed analysis of the positive, low-prevalence herds revealed that the majority of infected animals are in older age cohorts (see Fig. 5). In these herds targeted replacement strategies could result in BoHV-1 clearance without vaccination efforts.

### Sampling strategy

The study's sampling strategy, although comprehensive, may have certain implications on the interpretation of apparent prevalence rates. The herds surveyed in the NBWS appear to be a representative subset of all beef breeding herds in Ireland in terms of herd type composition (see Fig. 1). However, there are some differences in herd size distribution between NBWS herds and the overall population of beef herds (see Fig. 2). The overrepresentation of larger herds in the NBWS sample, with a notable underrepresentation of small herds (0–20 animals), could potentially skew the reported herd-level prevalence upwards. Within this study, we have seen that the level of infection is higher in larger herds. Consequently, the actual herd-level prevalence in the national beef herd might be lower than the reported 48.8% in the NBWS sample. Moreover, the overrepresentation of older animals in the tested group could influence the animal-level prevalence estimate, given that age is a known risk factor for BOHV-1 seropositivity [11, 22]. While the study adhered to guidelines recommending sampling animals over 9 months of age, this age bias should be acknowledged when interpreting the estimated animal-level prevalence of 11.4%.

In this study a'snapshot'testing strategy was employed to assess the herd-level BOHV-1 antibody status. This involved testing a random sample of up to 20 animals from each of the 10,650 beef breeding herds participating in the NBWS. If the NBWS testing approach were extrapolated to the national herd, approximately 27% of beef herds would undergo full herd testing through the snapshot, by virtue of having 20 or fewer animals, with a high proportion anticipated to return negative results.

### Multivariable risk factor analysis

The multivariable risk factor analysis identified two significant factors associated with recent BoHV-1 circulation, reaffirming findings from previous studies. Herd size and the number of in-moves per capita emerged as the most influential risk factors, aligning with existing literature on BoHV-1 epidemiology [12, 21, 22]. Larger herds and those with higher per capita rates of animal movements exhibited increased odds of recently experiencing active virus circulation, emphasizing the role of herd management practices and herd dynamics in disease transmission. These results underscore the importance of targeted interventions focusing on herd size management and biosecurity measures to mitigate the spread of BoHV-1 within the Irish beef cattle sector.

This study also revealed notable geographic variation in BoHV-1 herd-level prevalence across counties, with values ranging from 35.7% in Monaghan to 63.1% in Carlow. While these differences are clearly visualised in the results, a detailed exploration of underlying drivers, such as regional variation in vaccination uptake, cattle density, biosecurity practices or interactions with other control programmes was beyond the scope of this study.

### Limitations

While this study provides valuable insights into the prevalence and risk factors of BoHV-1 in Irish beef herds, it is essential to acknowledge certain limitations. A key limitation of the study arises from the voluntary nature of the NBWS programme. As participation was optional, there is potential for self-selection bias in the dataset. More proactive or better-managed farms may have been more likely to engage in the scheme, possibly skewing prevalence estimates. Another limitation is the uncertainty surrounding the within-herd sampling methodology. Although guidance was issued to encourage random selection, all sampling was performed by local practicing veterinarians, and none of the study authors were directly involved. Therefore, it is possible that convenience sampling occurred in some herds, with veterinarians selecting animals that were easier to access or handle. Another limitation of this study is the exclusion of approximately 40% of participating herds due to incomplete test results. While the decision to include only herds with complete or nearly complete data (maximum one missing value) was made to ensure reliable herd-level classification, this may introduce selection bias.

Another limitation concerns the age of animals sampled in smaller herds. While the sampling protocol aimed to exclude animals under 9 months of age to avoid interference from maternally derived antibodies (MDA), this age threshold could not always be upheld in herds with fewer than 20 animals. In such cases, all animals present were sampled, irrespective of age. This may have introduced bias, as the presence of MDA in younger animals can yield false-positive results, thereby potentially inflating prevalence estimates in smaller herds.

Notably, the study lacks information on vaccine usage within the participating herds. This omission is significant, as it prevents the determination of whether vaccination strategies were employed in specific herds and, if so, how they were implemented, and their impact. The absence of this information may influence the interpretation of the results, as vaccinated herds may exhibit different seroprevalence patterns compared to non-vaccinated herds. However, the seropositive results in our study are due to exposure to the wild virus (gE) rather than vaccination, given that only marker vaccines, which allow differentiation between wild virus exposure and vaccination, have been permitted in Ireland since 2002 [18]. Future studies should consider incorporating data on vaccine use to provide a more comprehensive understanding of BoHV-1 dynamics in Irish beef herds.

A further limitation of the risk factor analysis is the absence of several potentially important confounding variables. Data on biosecurity practices, participation in other animal health programmes or detailed farm-level management were not available. These factors could influence BoHV-1 exposure risk and may confound the observed associations, particularly with respect to herd size and movement patterns.

The method used to infer recent BoHV-1 circulation in herds—based on the age of the youngest homebred seropositive animal—relies on the assumption that infection spreads uniformly across the herd and that antibody-positive animals represent past exposure. However, within-herd transmission may not be complete, especially in low-contact systems or herds with partial vaccination histories. While the age-based threshold offers a practical proxy for recent circulation, it may misclassify some herds, particularly in cases of incomplete transmission.

Finally, interaction terms between predictors were not included in the multivariable logistic regression model. The decision to exclude interaction terms was based on the exploratory nature of this analysis, the large number of potential variables, and the aim to maintain model interpretability.

## Conclusion

BoHV-1 remains a significant concern within the Irish beef cattle sector. This study, conducted under the NBWS, provides a comprehensive analysis of the prevalence and risk factors associated with BoHV-1 infection in Irish beef herds. The findings highlight a herd-level prevalence of 48.8% and an animal-level prevalence of 11.4%, indicating that BoHV-1 is endemic in the population. While these prevalence rates reflect a reduction from previous studies, they underscore the persistent challenge posed by the disease. The higher case density observed in larger herds and those with frequent animal in-moves emphasizes the need for targeted interventions. Specifically, herds with evidence of recent BoHV-1 circulation should be encouraged to implement vaccination strategies to reduce transmission risk and prevent reactivation. Conversely, BoHV-1-negative herds may benefit from enhancing general biosecurity measures (e.g., controlling visitor access, quarantine of incoming animals) to maintain their free status and reduce the likelihood of virus introduction.

## Data Availability

No datasets were generated or analysed during the current study.
